# Clinical Characteristics and Outcomes following Percutaneous Coronary Intervention in Unprotected Left Main Disease: A Single-Center Study

**DOI:** 10.3390/diagnostics13071333

**Published:** 2023-04-03

**Authors:** Ștefan Dan Cezar Moț, Adela Mihaela Șerban, Alexandru Achim, Alexandra Dădârlat-Pop, Raluca Tomoaia, Dana Pop

**Affiliations:** 1 Cardiology Department, Heart Institute Niculae Stăncioiu, 19-21 Motilor Street, 400001 Cluj-Napoca, Romania; 2 5th Department of Internal Medicine, Faculty of Medicine, “Iuliu Hatieganu” University of Medicine and Pharmacy, 400012 Cluj-Napoca, Romania; 3 Cardiology Department, Kantonsspital Baselland, 4410 Liestal, Switzerland; 4 Department of Cardiology, Clinical Rehabilitation Hospital, 400347 Cluj-Napoca, Romania

**Keywords:** left main, percutaneous coronary intervention, stent enhancement, stent apposition, side branch, POT, kissing, bifurcation

## Abstract

**Background**: Hemodynamically significant unprotected left main (LM) coronary artery disease is a high-risk clinical condition because of the large area of myocardium at risk, and it requires prompt revascularization. Percutaneous coronary intervention (PCI) is an appropriate alternative to coronary artery bypass grafting (CABG) for revascularization of unprotected LM disease in patients with low-to-intermediate anatomic complexity or when the patient refuses CABG after adequate counseling by the heart team. **Methods**: We retrospectively evaluated 201 patients receiving left main (LM) provisional one-stent or two-stent procedures, and we assessed the clinical characteristics and outcomes of patients undergoing unprotected LM PCI. **Results**: The mean age was 66.5 ± 9.9 years, and 72% were male. The majority of the subjects presented several cardiovascular risk factors, among which arterial hypertension (179 patients, 89.5%) and dyslipidemia (173 patients, 86.5%) were the most frequent. Out of all patients, 162 (81.8%) underwent revascularization by using the one-stent technique, while the two-stent technique was used in 36 patients (18.2%). The median value of fractional flow reserve (FFR) of the side branch was 0.9 [0.85–0.95], and 135 patients (67.1%) showed a value of FFR > 0.8. One hundred nine patients (54.2%) had a stent enhancement side branch length (SESBL) > 2, with median values of 2.5 mm^2^ [2.1–3]. Regarding angiographic parameters, the LM area as assessed by intravascular ultrasound (IVUS) or optical coherence tomography (OCT) and the grade of stenosis as assessed by quantitative coronary angiography (QCA) were similar between groups. However, patients who required revascularization by using the two-stent technique presented more frequently with intermediate rather than low SYNTAX scores (69.4% vs. 28.4%, *p* < 0.0001). Also, the same group required kissing balloon inflation (KBI) more frequently (69.4% vs. 30%, *p* < 0.001). There were no differences regarding the success of revascularization between the use of the one-stent or two-stent technique. FFR was able to predict a SESBL > 2 mm. The cut-off value for FFR to afford the highest degree of sensitivity (74.5%) and specificity (47%) for a SESBL > 2 was >0.86, indicating a moderate accuracy (AUC = 0.61, 95% CI 0.525–0.690, *p* = 0.036). **Conclusions**: Unprotected left main PCI is a safe and effective revascularization option amongst a complex and morbid population. There were no differences regarding the success of revascularization between the use of the one-stent or two-stent technique, and there was no significant impact of KBI on side branch FFR measurements but lower side branch FFR values were correlated with angiographic side branch compromise.

## 1. Introduction

Patients with left main (LM) coronary artery stenosis are classified into two subgroups: protected (a previous patent coronary artery bypass surgery graft to one or more major branches of the left coronary) and unprotected LM (without such bypasses). Although coronary artery bypass grafting (CABG) is considered the gold standard of revascularization, technical improvements and stent technology made percutaneous coronary intervention (PCI) an increasingly utilized method of revascularization in patients with unprotected LM artery disease [1,2,3]. Complex PCI, including unprotected left main, is being performed at some centers where there is no on-site surgery, with no increase in major cardiovascular events or emergency CABG surgery compared with PCI at surgical centers, and a recent expert consensus statement supports the safety of PCI in this setting [1]. Patients with an obstruction of the LM may be at particularly high risk due to its anatomical features because LM provides 75–100% of the myocardium depending on coronary artery dominance [2]. With the introduction of revascularization procedures, the poor prognosis of individuals with LM coronary artery disease gradually improved. When compared to non-LM coronary artery disease, significant unprotected LM coronary artery disease is frequently associated with severe multivessel disease and higher mortality and morbidity, occurring in 3–5% of patients with coronary artery disease and being the subject of continuous investigation [3,4,5].

Recent studies and meta-analyses demonstrated that PCI in this lesion subset is a feasible alternative offering similar results when compared with surgical revascularization [6,7,8]. However, these trials have indicated a significant time-dependent treatment interaction, with the early advantage of PCI in terms of peri-procedural myocardial infarction (MI) and stroke being offset by a greater risk of spontaneous MI compared to CABG during long-term follow-up [6,7,8]. Outcomes were improved on both sides, but there are still many debates on some interventional aspects. One of the pivotal randomized controlled trials directly comparing CABG with PCI with stenting was the NOBLE (Nordic-Baltic-British Left Main Revascularization Study) trial. [9]. This study revealed more adverse cardiovascular and clinical outcomes with PCI than CABG due to higher revascularization rates, especially in patients with a high Syntax Score (>32), and these findings were confirmed at a 5-year follow-up [9]. The 5-year follow-up of NOBLE by Holm and colleagues found that PCI increased MI and repeat revascularization, but not all-cause mortality, compared with CABG [9]. These data suggest that an increased coronary atherosclerotic burden may be associated with impaired clinical outcomes in patients with unprotected LM disease after PCI. It may be related to increased residual CAD severity after revascularization of the LM, or in fact, it may be related to outcomes directly related to the operator and the quality standard of the PCI steps.

The evidence thus suggests that PCI is a reasonable treatment alternative for this subset of coronary artery disease patients, although at the cost of a higher rate of target lesion revascularization. This theoretical higher rate of target lesion revascularization may be explained by two factors: PCI-related factors and patient-related factors.

Regarding the PCI-related factors, a three-level decision-making process is mandatory in LM PCI. First, in cases of angiographic ambiguity, intravascular imaging and fractional flow reserve can be employed to determine whether revascularisation can be deferred. Second, if revascularisation is necessary, the risks and benefits of percutaneous vs. surgical methods should be weighed. Third, if PCI is adopted, the operator must choose between a provisional single-stent strategy and an upfront two-stent strategy. Regardless of the PCI approach used, it should be carried out in accordance with the guidelines of a stepwise procedure that includes proximal optimization (POT) after each instance of crossover stenting and kissing balloon inflation (KBI) as needed.

Yet, the clinical characteristics of these patients are equally important in determining success after PCI. These patients are often complicated due to advanced age, higher frailty scores, complex and more severe coronary anatomy, worse left ventricular systolic dysfunction (LVSD), and the presence of more comorbid conditions. Inevitably, their outcomes may differ. In conclusion, benefiting from a tertiary center with frequent and standardized LM revascularizations (few and experienced operators), we performed a retrospective analysis of these procedures.

The aim of this study was to investigate the clinical characteristics and outcomes of patients undergoing unprotected LM PCI in a high-volume PCI center, with emphasis on patient profile, stenting techniques (1 stent vs. 2 stents) and the clinical relevance of side branch compromise (residual stenosis measured by quantitative coronary angiography attributed to the stent enhancement side branch length [SESBL] sign or fractional flow reserve) to the final procedural success.

## 2. Materials and Methods

### 2.1. Study Population

This research was conducted in accordance with the Declaration of Helsinki and was approved by the Ethics Committee of “Nicolae Stăncioiu” Heart Institute, Cluj-Napoca, Romania, number 17/2017. The study was a retrospective, single-center, observational study of patients with LM lesions who underwent LM bifurcation PCI. Between January 2019 and December 2020, a total of 201 patients were consecutively enrolled from 1 tertiary, high-volume cardiac center. All patients offered their written informed consent. Inclusion criteria were patients with angiographic evidence of a significant bifurcation lesion (stenosis of at least 70% diameter at 1, 2 or both branches) and clinical indication for PCI with stent implantation. Exclusion criteria included contraindication for PCI or PCI without stent implantation, prior revascularization with a stent at the level of LM, more than mildly reduced left ventricular ejection fraction on admission and severe comorbidities (severe renal failure, frailty, non-cardiac comorbidities) that deferred a high-risk PCI.

All patients underwent coronary artery angiography with either the 1-stent or 2-stent revascularization. Post-stenting FFR was measured at the level of the side branch (SB) in the majority of patients. The angiography that recommended the revascularization procedure was analyzed and used for the calculation of the Syntax II score in order to classify the patients as low- (≤22) or intermediate-risk (23–32). The severity of the LM stenosis was quantified using the visually assessed diameter of stenosis > 70% or by using FFR ≤ 80% in case of a 50–70% stenosis, which was followed by the calculation of QCA of the LM and of LM area using IVUS or OCT.

### 2.2. The PCI Procedure

LM coronary artery revascularization was performed in all patients. PCI was performed by using solely drug-eluting stents (DES). Baseline characteristics, symptomatic status, number of diseased vessels per patient and bifurcation disease were assessed. The procedure was performed by only experienced interventionalists (more than 50 LM PCIs per year). All decisions regarding material, device selection or the appropriate treatment strategy were left to the treating physician. Generally, the LM and left anterior descending (LAD) artery were usually regarded as the main branch, and the left circumflex (LCX) artery was regarded as SB. IVUS, OCT or FFR was conducted at the level of the bifurcation at the discretion of the responsible operator. POT and stent enhancement was performed in all patients. Patients were recruited irrespective of performing KBI.

When considering the optimal strategy for LM revascularization, not only the severity of CAD and the possibility of achieving complete revascularization is important, as comorbidities, age, and past medical history also influence the treatment. The factors that favored the LM PCI decision were: advanced age, comorbidities, high surgical risk, frailty, unfavorable anatomy for surgical revascularization, reduced life expectancy, restricted mobility, ostial/shaft lesion, SYNTAX score < 23 points, urgent revascularization, patient preference. During the index procedure, the operator decided whether to use the 1-stent or 2-stent technique for an LM lesion based on the bifurcation classification, the coronary flow of the main and side branches, angulation, vessel dominance, and calcification. Except in situations where balloon or wire passing failed, the final kissing procedure of LM bifurcation following stenting was an essential step in all 2-stent cases. All additional non-LM lesions were treated in the same manner with the goal of achieving full revascularization. The hospital database’s medical records were evaluated retrospectively for statistical analysis of baseline demographic data and in-hospital and long-term outcomes.

All the participants received medical treatment after the procedure according to current recommendations. Aspirin was administered lifelong in both groups, and a P2Y12 inhibitor was associated after PCI for 6–12 months, according to the type of clinical presentation of admission. Intra- and post-procedural complications were noted, and LVEF was calculated before and at 48 h after PCI.

Angiographic success was defined as 30% LM residual stenosis, 3 mm minimal lumen diameter, flow grade 3 Thrombolysis in Myocardial Infarction, and no dissection. The use of intravascular imaging to confirm angiographic success was highly encouraged. Clinical success was defined as angiographic success as well as death/myocardial infarction/stroke-free in-hospital outcome.

### 2.3. Definitions

Unprotected left main stenosis was defined as LMCA stenosis without previous history of CABG or absence of patent grafts in an angiogram if previous CABG had been done. Significant LM stenosis was defined as angiographic diameter stenosis > 50%.

Provisional stenting was defined as stenting across the main vessel, followed by side branch stenting if required. The 2-stent strategy was defined as upfront stenting of both the main vessel and side branch with any of the standard techniques according to the bifurcation anatomy and operator experience.

Procedural success was defined as post-procedure TIMI grade 3 flow and residual stenosis < 30%; the clinical success of the intervention was defined as freedom from death or the need for urgent revascularization within 24 h of the index PCI.

Target lesion revascularization was defined as any repeat revascularization (PCI or CABG) for restenosis inside the implanted stent or within 5 mm distal or proximal to the stent edges.

Stent enhancement side branch length (SESBL) was an additional marker of procedural success, representing optimal stent apposition at the level of the polygon of the confluence of the LM. SESBL was defined as the translucent area measured in millimeters during stent enhancement acquisitions at the level of the side branch. For an accurate measurement, all coronary angiograms were reviewed and analyzed blindly by 2 investigators. A prespecified measuring protocol was defined, and SESBL was considered as the translucent length measured at the level of SB, overlapping the Quantitative Coronary Analysis (QCA) over the stent enhancement images. The software was calibrated with the diameter of the catheter, and then the investigator could draw the contour of the SESBL. The final SESBL length represented the average between the 2 measurements. For precision, the diameter of the stent (which was already known) was also verified with QCA.

As previously stated, intracoronary imaging was encouraged, and the procedural quality metrics were the following:

- For IVUS: minimum lumen cross-sectional area in a stented segment > 5.0 mm^2^ or 90% of the distal reference lumen cross-sectional area, plaque burden at the 5 mm proximal or distal to the stent edge < 50% and no edge dissection involving the media > 3 mm in length;
- For OCT: proximal mean stent area > 90% of the proximal reference vessel minimum lumen area, distal mean stent area > 90% of the distal reference vessel minimum lumen area, full stent apposition (no more than >3.0 mm from the vessel wall for longer than 3mm of the vessel) and no dissection that penetrates the media and >90° in an arc.

### 2.4. Statistical Analysis

Clinical variables were expressed as mean ±SD/median [IQR] or frequencies depending on the type and distribution. Normality was tested via the Kolmogorov–Smirnov test. Patients were divided into 2 groups according to the use of the 1- or 2-stent technique. Continuous data were compared using *t*-tests if there was a normal distribution or Mann–Whitney test otherwise, and categorical data by using the Chi2 test or Fisher’s test. Differences between revascularization techniques according to the parameters used to evaluate the success of the procedure (FFR > 0.8 or SESBL > 2 mm) were assessed by using the Chi2test. The relationship between each technique (1- vs. 2-stent technique, kissing-balloon inflation, POT) and each of the success criteria (FFR, SESBL) was evaluated by using the regression analysis. Results were depicted by using the corresponding scatter diagrams and by reporting the obtained regression equation, coefficients of determination R^2^, 95% CI and the *p*-value of intercept. Logistic regression was used to determine if a model incorporating 1- or 2-stent technique, kissing-balloon inflation, and POT may determine the success of revascularization, as assessed by FFR > 0.8 or SESBL > 2. The model was tested by using both the enter and backwise methods. Analysis receiver operating curves (ROC) were performed to assess the sensitivity and specificity of (1) FFR to predict a value of SESBL > 2 and (2) SESBL to predict a value of FFR > 0.8. Statistical analysis was performed with MedCalc Statistical Software 19.6.1 (MedCalc Software Ltd., Ostend, Belgium; http://www.medcalc.org (accessed on 30 November 2022)). A *p*-value of <0.05 was considered significant.

## 3. Results

The studied population included 201 patients with 234 stents deployed using one (“provisional”) or two stents technique for LM bifurcation stenosis. Clinical data of the patients are shown in Table 1. The mean age was 66.5 ± 9.9 years, and 72% were male. The majority of the subjects presented several cardiovascular risk factors, among which arterial hypertension (179 patients, 89.5%) and dyslipidemia (173 patients, 86.5%) were the most frequent.

Many bifurcations had lesions in both the main vessel (proximal and distal segments) and side branch, classified as Medina 1,1,1 or true bifurcation lesion (n = 105, 52%) and the rest included less diseased bifurcations that allowed provisional one-stent technique PCI. Regarding angiographic parameters, the LM area, as assessed by IVUS or OCT, showed values of 3.93 ± 2.8 mm^2^, while the grade of stenosis, as assessed by QCA, was 82.5 ± 10.8%. Out of all patients, 162 (81.8%) underwent revascularization by using the one-stent technique, while the two-stent technique was used in 36 patients (18.2%). KBI was used in 74 of all subjects (36.8%) and POT in 198 patients (98.5%). FFR in the SB was used in approximately a quarter of the patients (n = 51, 25%), and the overall intracoronary imaging (IVUS or OCT) usage rate was 40% (n = 79). Results were quantified by using FFR and the SESBL. Median values of FFR of the side branch were 0.9 [0.85–0.95], and 135 patients (67.1%) showed a value of FFR > 0.8. One hundred nine patients (54.2%) had a SESBL > 2, with median values of 2.5 mm^2^ [2.1–3]. In the case of FFR < 0.8, an LM two-stent technique was performed.

LVEF before PCI was 49.1 ± 7.9%, while the post-PCI values at 48 h were 49.6 ± 10. 10.1%. Regarding the monitoring period after PCI, 3% of the patients required inotropic/vasopressor support, while no patients developed the necessity of IABP.

Clinical data of the patients in accordance with the revascularization technique used (one- vs. two-stent) are depicted in Table 2. There was no difference in age, gender, type of coronary syndrome on admission (acute vs. chronic), or risk factors between groups.

Regarding angiographic parameters, the LM area as assessed by IVUS or OCT and the grade of stenosis as assessed by QCA were similar between groups. However, patients who required revascularization by using the two-stent technique presented more frequently with intermediate rather than low SYNTAX scores (69.4% vs. 28.4%, *p* < 0.0001). Also, the same group required KBI more frequently (69.4% vs. 30%, *p* < 0.001). There were no differences regarding the success of revascularization between the use of the one-stent or two-stent technique; the two groups showed similar values for side branch FFR (0.9 vs. 0.89, 68.5% vs. 66.6% patients with FFR > 0.8) and for the SESBL (2.5 vs. 2.55, 56.2% vs. 47.2% patients with SESBL > 2 mm).

The LVEF, both before and after PCI, did not significantly differ among groups. Also, the percentage of patients requiring inotropic/vasopressor support was similar between groups.

The differences between revascularization techniques according to the parameters used to assess the success of the procedure are shown in Table 3. There was no significant difference regarding the use of either revascularization technique between groups according to FFR or SESBL, except for the KBI, which was more frequently used in the group with successful side branch revascularization (FFR > 0.8)—42.2 vs. 25.8%, *p* = 0.023. There was a significantly higher Syntax Score in the two-stent technique group.

**Figure 1:**
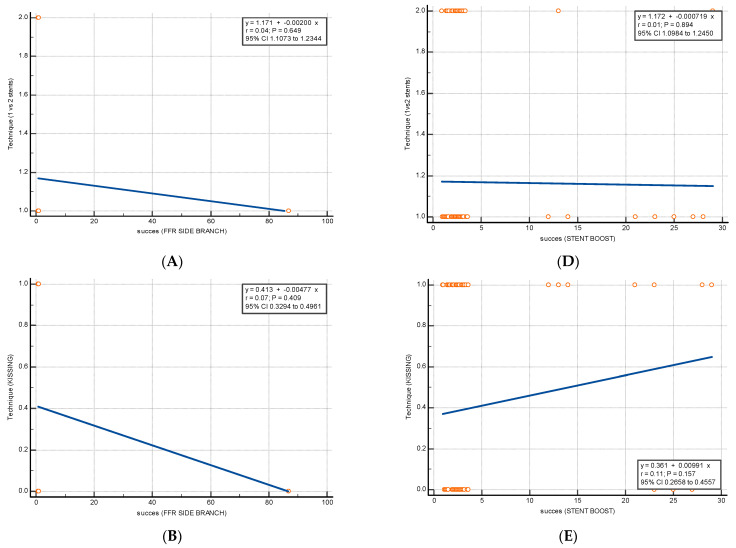

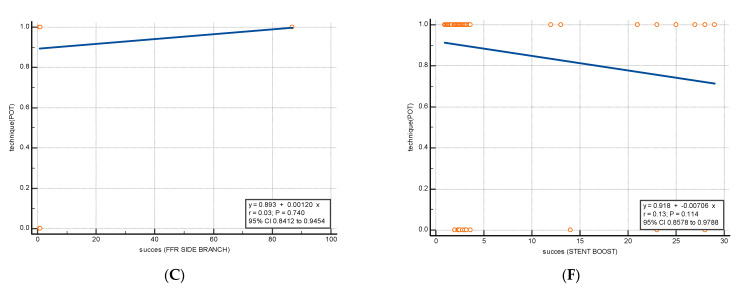
Regression analysis for each technique and success criteria. (**A**) Technique—one- vs. two-stent technique; success—FFR side branch > 0.8 (Standard error of the slope = 0.004, p = 0.65). (**B**). Technique—kissing-balloon inflation; success—FFR side branch > 0.8 (Standard error of the slope = 0.006, p = 0.41). (**C**) Technique—POT; success—FFR side branch > 0.8 (Standard error of the slope = 0.0036, p = 0.74). (**D**). Technique—one- vs. two-stent technique; success—SESBL > 2 (Standard error of the slope = 0.0053, p = 0.89). (**E**) Technique—kissing-balloon inflation; success—SESBL > 2 (Standard error of the slope = 0.0069, p = 0.16). (**F**) Technique—POT; success—SESBL > 2 (Standard error of the slope = 0.0044, p = 0.12). FFR, fractional flow reserve; POT, proximal optimization technique.

The regression analysis and the corresponding scatter diagrams used to evaluate the relationship between each technique (one- vs. two-stent technique, kissing-balloon inflation, POT) and each of the success criteria (FFR and SESBL) are depicted in Figure 1. Neither of the revascularization techniques was associated with a better chance of successful revascularization.

By using logistic regression, we tested if a model incorporating the one- or two-stent technique, kissing-balloon inflation and POT may determine the success of revascularization, as assessed by FFR > 0.8 or SESBL > 2. The results are depicted in Table 4. Neither model demonstrated an association with a better chance of revascularization (FFR-*p* = 0.14, SESBL-*p* = 0.74). However, the use of KBI was the only technique able to predict an FFR > 0.8 (OR = 2.2, 95% CI = 0.304–2.389, *p* = 0.027), thus successful revascularization. However, KBI was not also able to predict a SESBL of >0.2 mm (*p* = 0.66).

**Figure 2:**
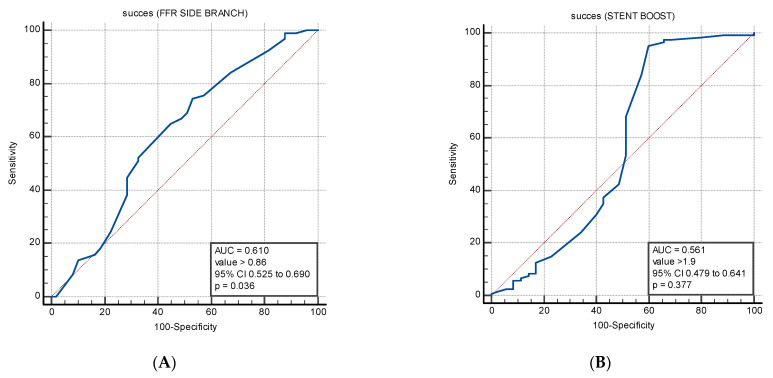
ROC curves showing the prognostic sensitivity and specificity of (**A**) FFR values to predict a value of SESBL > 2. The AUC of FFR (0.61, 95% CI 0.525–0.690, p = 0.036) with the optimal cut-off value of >0.86, which provided Se = 74.5% and SP = 47% for the prediction of SESBL values > 2. (**B**) SESBL values to predict a value of FFR > 0.8. The AUC of SESBL (0.56,95% CI 0.479–0.641, p = 0.38) with the optimal cut-off value of >1.9, which provided Se = 95% and SP = 40% for the prediction of FFR values > 0.8.

The ROC curves showing the sensitivity and specificity of FFR to predict a value of SESBL > 2 mm are shown in Figure 2A, while the ROC curves showing the sensitivity and specificity of SESBL values to predict a value of FFR > 0.8 are shown in Figure 2B.

Out of the two parameters, FFR was the only one able to predict a SESBL > 2 mm. The cut-off values for FFR to afford the highest degree of sensitivity (74.5%) and specificity (47%) for a SESBL > 2 were >0.86, indicating a moderate accuracy (AUC = 0.61, 95% CI 0.525–0.690, *p* = 0.036).

Regarding the diameter assessed by stent-boost, although statistically insignificant (*p* = 0.38), a cut-off value of >1.9 mm predicted with high sensitivity (95%) and moderate accuracy (AUC = 0.56, 95% CI 0.479–0.641), a value of FFR > 0.8.

A short-term follow-up was assessed in all patients, either via telephone or during the outpatient clinic visits, and no difference in major cardiovascular events (cardiac death, myocardial infarction, target lesion failure or target lesion revascularization) was observed between the two groups (overall MACE n = 6, 3.6% in the one-stent technique group vs. n = 2, 5% in the two-stent technique group, *p* = 0.12).

## 4. Discussion

The major findings from the present study, which investigated the clinical and procedural role of PCI in modern LM revascularization, were the following: (1) there were no differences regarding the success of revascularization between the use of one-stent or two-stent technique, (2) there was no significant impact on the FFR side branch measurements if whether KBI was performed or not (3) lower side branch FFR values were correlated with lower values of stent apposition lengths measured by quantitative coronary angiography at the level of the side branch (SESBL < 2.0 mm) and the use of KBI was the only technique able to predict an FFR >0.8, thus demonstrating that KBI may indeed improve the flow towards the side branch. A shorter SESBL implies an underexpanded stent at the level of the side branch. Oftentimes, at the polygon of confluence, the distal main vessel stent is not fully “deformed” by POT for two main reasons: the previously mentioned too-proximal POT or because plaque burden and distribution is frequently found at the distal LM shoulder. Physiologically, the diameter of the polygon of confluence is larger than the rest of the LM. The previously described “melon seed” effect of the POT balloon in a funnel-shaped LM could push out the POT balloon toward the aorta [10]. This could be prevented by adequate lesion preparation.

Shorter SESBL translates into underexpansion at this level, an aspect that could be seen and further improved with IVUS or OCT. The fact that KBI did not influence the clinical outcomes is also in concordance with other studies [11,12,13]; as with provisional stenting, the benefit of using KBI could indeed provide better flow dynamics, eliminate floating struts and restore the anatomical shape of the bifurcation but in the end, an optimally performed POT could almost remove stent struts from the SB emergence [14].

When performing unprotected LM PCI, the following technical considerations are important: (i) patient comorbidities: elderly age, diabetes, renal failure, acute coronary syndrome on presentation, left ventricular dysfunction, concomitant valvular disease, previous cerebrovascular events, etc.; (ii) lesion morphology: lesion location—ostial, shaft or bifurcation, presence of calcification, angulation, smaller LM diameter, associated multivessel disease, presence or absence of the patent right coronary artery and collaterals to the left system, the dominance of left circumflex; (iii) use of additional equipment: to optimize and safely accomplish the LM intervention, consideration must be given to the use of additional equipment such as intravascular imaging, physiologic assessment, mechanical circulatory support, and ventilatory support. Vascular access is also important, and radial access is recommended even for larger catheters [15], with the novel distal radial access having already been proven to support sheaths up to 8-French [16,17].

Our study’s findings are consistent with the current data on unprotected LM PCI. POT is a mandatory step during the intervention. In practical terms, adapting the stent contour to the underlying fractal anatomy of a bifurcation implies adjusting for malapposition and/or underexpansion in the proximal section (Figure 2). Aside from the immediate procedural repercussions of failing to perform POT, such as abluminal side branch rewiring and its accompanying risks, current clinical evidence suggests that POT can help reduce the rate of target lesion failure in a large all-comer registry of patients having bifurcation PCI [18]. A poor outcome in the SB following POT may demand further intervention; however, routine KBI after main artery stenting has not been demonstrated to enhance outcomes in a randomized trial involving non-LM bifurcations [19]. Observational evidence in patients with distal LM bifurcation lesions treated with the provisional approach confirmed that final KBI has no benefit or harm when used routinely [20,21]. When performed as part of a two-stent bifurcation PCI, the mechanics of KBI, such as bringing back to the center the carina and overexpansion at the point of confluence and in the proximal main vessel, appear to offer the highest therapeutic benefits [22]. Although a recent sub-analysis of the EXCEL trial found no benefits of final KBI in patients treated with one or two stents [23], there is compelling both historical and current evidence [24] that validates the value of final KBI when a bifurcation is treated with two stents, and it is now recognized as a procedural quality indicator when performing two-stent bifurcation PCI [25].

Our study revealed significantly lower FFR values in SESBL < 2.0 mm and KBI was more frequently used in the group with successful side branch revascularization (FFR > 0.8), and these findings are clinically relevant as well. Provisional stenting may cause angiographic side branch jailing by the mechanism of carina or plaque shift. We demonstrated that, in this case, FFR measurement in the jailed side branch could be useful in evaluating hemodynamic impairment in the LCX branch, thus reducing the need for extra complex procedures. Moreover, Lee et al. demonstrated that patients with a high FFR in the jailed LCX had better 5-year target lesion failure (a composite of cardiac death, target vessel MI, or target lesion revascularization) outcomes than those with a low FFR [26]. Of course, this is dependent on the magnitude of the diameter of the side branch. In contrast, Peng et al. found no difference in terms of major adverse events when not treating the side branch, but their cohort included small side branches only [27]. Nevertheless, periprocedural MI always occurs in the setting of side branch occlusion, which may result in significant adverse clinical events [28,29].

In a study describing the utility of stent enhancement (StentBoost) to guide PCI for bifurcation lesions, Da Silva et al. intuitively stated 10 years ago that frequently, angiographic images alone do not permit adequate visualization of stent deformation or incomplete stent expansion at the ostium of the side branch and stent enhancement techniques may help in this regard [30]. With current live stent enhancement techniques, technological advances in this particular domain are on the horizon [31]. The steps of LM PCI can be furthermore accurately controlled, especially since intracoronary imaging cannot give live insights while positioning gear inside the coronary arteries. Most importantly, the optimal positioning of the POT balloon can be guided by the contrast injection during live stent enhancement visualization and maneuvering [32,33]. The main advantages of stent enhancement over intracoronary imaging are its simplicity of use, short time requirements, lack of additional costs, and immediate image interpretation [34]. Numerous minor studies comparing stent enhancement to IVUS found a good correlation between the two approaches for measuring minimal stent diameter [35,36,37]. Since stent enhancement techniques were often used in our study and intracoronary imaging techniques were used relatively little, our results also contribute in this regard, having the largest study population so far.

The study has several limitations worthy of mentioning. This was a single-center, nonrandomized, observational study, and although the sample size was relatively large, these results need to be validated by further studies. Second, the choice or assignment of revascularization modality in the present study was based on the attending physician’s recommendation, the patient’s and family’s choice and their financial capability rather than random assignment. However, we did not have significant differences in comorbidities between the groups at the study entry. Third, the SESBL is subjective by the clarity of the stent enhancement visualization and by the operator who performs the measurement; nevertheless, we aimed for an objective SESBL measuring protocol. A signal-to-noise ratio is reduced in heavily calcified vessels and segments with multiple stents, although in bifurcations, there is the problem of overlap of radiopaque structures with a proper view selection.

## 5. Conclusions

Unprotected left main PCI is a safe and effective revascularization option amongst a complex and morbid population. A higher Syntax Score was correlated with performing a two-stent PCI technique. There were no differences regarding the success of revascularization between the use of the one-stent or two-stent technique, and there was no significant impact of KBI on side branch FFR measurements but lower side branch FFR values were correlated with angiographic side branch compromise. These findings reassure the importance of KBI and the use of two-stent techniques when the angiographic or the physiologic data demands it.

## Figures and Tables

**Table 1:** General characteristics of the patients.

Variable	Value
Age, years (mean ± SD)	66.5 ± 9.9
Male gender (n,%)	145 (72)
**Risk factors**	
Arterial hypertension (n,%)	179 (89.5)
Obesity (n,%)	58 (29)
Smoking (n,%)	29 (14.6)
Dyslipidemia (n,%)	173 (86.5)
Diabetes (n,%)	75 (37.5)
COPD (n, %)	26 (12.9%)
Atrial fibrillation	44 (21.8%)
**Preprocedural characteristics**	
LM area IVUS/OCT, mm^2^ (mean ± SD)	3.93 ± 2.8
QCA–LM, % (mean ± SD)	82.5 ± 10.8
LVEF before PCI (mean ± SD)	49.1 ± 7.9
**Intraprocedural characteristics**	
Number of patients with one-/two- stent technique (n,%)	162 (81.8)/36 (18.2)
Number of patients with Kissing balloon inflation (n,%)	74 (36.8)
Number of patients with POT (n,%)	198 (98.6)
IVUS/OCT evaluation	79 (40)
FFR in the SB	51 (25.3)
FFR-side branch (mean ± SD) (median [IQR])	0.9 [0.85–0.95]
Number of patients with FFR > 0.8 (n,%)	135 (67.1)
SESBL, mm (median [IQR])	2.5 [2.1–3]
Number of patients with SESBL > 2 (n,%)	109 (54.2)
Intraprocedural complications (%)	19 (10.1)
**Postprocedural characteristics**	
Necessity of inotropic/vasopressor medication during hospitalization (n,%)	6 (3)
Necessity of IABP (n,%)	0 (0)
Number of days of admission (mean ± SD)	3.5 ± 1
LVEF after PCI (mean ± SD)	49.6 ± 10

FFR, fractional flow reserve; IABP, intra-aortic balloon pump therapy; IVUS, intravascular ultrasound; LM, left main; LVEF, left ventricular ejection fraction; NSTEMI, non-ST elevation myocardial infarction; OCT, optical coherence tomography; PCI, percutaneous coronary intervention; POT, proximal optimization technique; STEMI, ST-elevation myocardial infarction.

**Table 2:** Differences between characteristics of the patients according to the one- or two-stent technique.

Variable	Patients with One-Stent Technique (n = 163)	Patients with Two-Stent Technique (n = 38)	*p* Value
Age, years (mean ± SD)	66.8 ± 9.9	65.3 ± 10.1	0.41
Male gender (n,%)	115 (71)	27 (75)	0.63
**Risk factors**			
Arterial hypertension (n,%)	147 (90.7)	36 (100)	0.06
Obesity (n,%)	47 (29)	10 (27.8)	0.87
Smoking (n,%)	21 (13)	7 (19.4)	0.33
Dyslipidemia (n,%)	137 (84.6)	33 (91.7)	0.30
Diabetes (n,%)	60 (37)	14 (38.9)	0.85
**Preprocedural characteristics**			
LM area IVUS/OCT, mm^2^ (mean ± SD)	3.81 ± 1.36	4.17 ± 0.94	0.16
QCA—LM (mean ± SD)	83.3 ± 10.7	80.4 ± 11	0.17
LVEF before PCI (mean ± SD)	49.4 ± 7.7	47.6 ± 9.14	0.22
**Intraprocedural characteristics**			
Number of patients with intermediate SYNTAX score (n,%)	46 (28.4)	25 (69.4)	<0.0001
Number of patients with kissing-balloon inflation technique (n,%)	49 (30)	25 (69.4)	<0.0001
Number of patients with POT (n,%)	147 (90.7)	30 (83.3)	0.19
FFR in the SB	39 (23.9)	12 (31.5)	0.44
FFR-side branch (mean ± SD) (median [average rank])	0.9 [73.9]	0.89 [62.8]	0.23
Number of patients with FFR >0.8 (n,%)	111 (68.5)	24 (66.6)	0.83
SESBL, mm (median [IQR])	2.5 [77.3]	2.55 [7.4]	0.91
Number of patients with SESBL >2 (n,%)	91 (56.2)	17 (47.2)	0.33
Intraprocedural complications (%)	14 (8.6)	5 (13.9)	0.79
**Postprocedural characteristics**			
Necessity of inotropic/vasopressor medication during hospitalization (n,%)	4 (2.4)	1 (2.7)	0.91
Necessity of IABP (n,%)	0 (0)	0 (0)	0
Number of days of admission (mean ± SD)	3.4 ± 3	4.2 ± 4	0.22
LVEF after PCI (mean ± SD)	49.8 ± 8.14	48.6 ± 16.3	0.55

FFR, fractional flow reserve; IABP, intra-aortic balloon pump therapy; IVUS, intravascular ultrasound; LM, left main; LVEF, left ventricular ejection fraction; NSTEMI, non-ST elevation myocardial infarction; OCT, optical coherence tomography; PCI, percutaneous coronary intervention; POT, proximal optimization technique; STEMI, ST-elevation myocardial infarction.

**Table 3:** Differences between revascularization techniques according to the parameters used to assess the success of the procedure.

	FFR > 0.8 (n = 135, 67.2%)	FFR ≤ 0.8 (n = 66, 32.8%)	*p*	SESBL > 2 (n = 109, 54.2%)	SESBL ≤ 2 (n = 92, 45.8%)	*p*
Number of patients with one-stent technique (n,%)	111 (82.2%)	51 (77.3%)	0.225	91 (83.5%)	71 (77.2%)	0.514
Number of patients with two-stent technique (n,%)	24 (17.8%)	12 (18.18%)		17 (15.6%)	19 (20.7%)	
Number of patients with kissing-balloon inflation (n,%)	57 (42.2%)	17 (25.8%)	0.023	38 (34.9%)	36 (39.1%)	0.533
Number of patients with POT (n,%)	120 (88.9)	58 (87.9%)	0.833	97 (89%)	81 (88%)	0.833

FFR, fractional flow reserve; POT, proximal optimization technique.

**Table 4:** Logistic regression analysis for each technique and FFR >0.8/SESBL >2.

Independent Variable	Covariate	Odds Ratio	95% Confidence Interval	*p* Value	Overall Model Fit (*p*)
FFR	One- vs. two-stent technique	0.6591	0.2849 to 1.5249	0.3300	0.14
	Kissing-balloon inflation	2.2018	1.0935 to 4.4334	0.0271	
	POT	0.8520	0.3038 to 2.3892	0.7607	
SESBL	One- vs. two-stent technique	0.7260	0.3378 to 1.5604	0.4122	0.74
	Kissing-balloon inflation	0.8748	0.4754 to 1.6100	0.6674	
	POT	0.8429	0.3357 to 2.1167	0.7160	

FFR, fractional flow reserve; POT, proximal optimization technique.

## Data Availability

Data is contained within the article or supplementary material. Additional data of this study are available on request from the corresponding author.

## Abbreviations

PCI	percutaneous coronary intervention	
CABG	coronary artery bypass grafting	
LM	left main	
POT	proximal optimization technique	
KBI	kissing balloon inflation	
QCA	qualitative comparative analysis	
SESBL	stent enhancement side branch length	
IVUS	intravascular ultrasound	
OCT	optical coherence tomography	
